# Upside-down in volcanic ash: crown reconstruction of the early Permian seed fern *Medullosa stellata* with attached foliated fronds

**DOI:** 10.7717/peerj.13051

**Published:** 2022-03-22

**Authors:** Ludwig Luthardt, Mathias Merbitz, Evgeny Fridland, Ronny Rößler

**Affiliations:** 1 Department of Diversity Dynamics, Museum für Naturkunde Berlin-Leibniz Institute for Evolution and Biodiversity Sciences, Berlin, Berlin, Germany; 2 Museum für Naturkunde, Chemnitz, Saxony, Germany; 3 Institute of Geology, TU Bergakademie Freiberg, Freiberg, Saxony, Germany

**Keywords:** Permian, Medullosales, Alethopterid foliage, Whole-plant concept, Plant architecture, Plant anatomy, Paleobotany, Late Paleozoic, Intramontane flora

## Abstract

Our understanding of fossil floras through geological time is mainly based on various differently preserved plant parts, often found isolated under restricted taphonomic circumstances. Preservation of whole plants is exceptionally rare in the geological record but provides the most reliable proof of ancient plants, especially those lacking a nearest living relative like the late Paleozoic pteridosperms. Among them, the medullosan seed ferns represent the largest and most essential systematic group. Medullosaleans are well known from the large Euramerican tropical swamp forests of Carboniferous age, but also from seasonally dry, clastic-soil habitats of Late Pennsylvanian–early Permian intramontane basins in central Europe. An exceptional taphonomic window that offered three-dimensional preservation of early Permian plants is located in Chemnitz, eastern Germany. Here, a forest ecosystem had been buried by pyroclastic deposits in a geological instant, 291 ± 2 Ma ago. Medullosaleans are both abundant and diverse in this striking autochthonous assemblage. The upper part of a *Medullosa stellata* var. *typica* individual broke at its top resulting from the overload of volcanic ash and was buried upside-down in the basal pyroclastics. The tree crown consists of the anatomically preserved apical stem, ten attached *Alethopteris schneideri* foliated fronds with *Myeloxylon*-type petioles and rachises. Though already discovered during the scientific excavation in 2010, the remarkable find required several years of preparation work, documentation, and reconstruction. The fronds were up to 3.5 m long, bifurcating, and bore numerous bipinnately compound pinnae preserved pulvinated in life position. The apical stem vascular system consists of a cylindrical peripheral vascular segment and up to 87 central accessory strands, each surrounded by manoxylic wood and secondary phloem. The reconstructed tree is supposed to have been self-supporting and of slender stature. Its architectural model is comparable to modern tree ferns or cycads and adapted to have grown in light-deficient lower-storey (sub-) tropical forests, usually not exceeding 15 m in height. Apical meristematic growth dominated, whereas only minor secondary growth occurred during ontogenesis. The densely attached frond bases followed a 3/8 phyllotaxis and were most likely abscised shortly after becoming photosynthetically inactive. A high water-conducting potential is assumed due to the tree’s cauline, petiolar and leaf vascular anatomies. Concerning the extensive leaf surface of the densely foliated fronds, considerable transpiration is hypothesised. *Alethopteris schneideri* foliage is stratigraphically significant for lower Permian (Asselian–Sakmarian) continental strata of central Europe, preferring habitats of wet clastic soils in sub-humid, seasonal palaeoclimate. The new insights provide a substantial step towards the first whole-plant concept of intramontane medullosaleans.

## Introduction

The late Paleozoic is a period of remarkable global change, characterised by a distinct shift of terrestrial ecosystem patterns and a major floral turnover (*e.g*., Kerp, 2000; DiMichele et al., 2006; Quie et al., 2019). A deep understanding of plant evolutionary processes and environmental change can only be achieved by a detailed knowledge of the important vegetational elements which together form different habitat types varying in structure, composition, and site-specific environmental conditions. Medullosans, a diverse group of pteridosperms, represent some of these characteristic vegetational elements widely distributed in forest ecosystems of the late Paleozoic era. This group of seed ferns is known from the late Mississippian to early Permian but probably occurred to the latest Permian (Dunn et al., 2003; Cotta, 1832; Kustatscher et al., 2014). Medullosan seed ferns had eustelic, slender parenchymatous stems with a specialised vascular system, encompassing cauline vascular strands with secondary tissues and, in some cases, central accessory strands (*e.g*., Basinger, Rothwell & Stewart, 1974; Mapes & Rothwell, 1980; Dunn, 2006; Luthardt et al., 2021). Attached to the stems were large fronds with petioles of the *Myeloxylon* type (*e.g*., Renault, 1875; Grand’Eury, 1877; Beeler, 1983), bearing neuralethopterid, neurodontopterid and cyclopterid foliage (*e.g*., Laveine, 1997; Cleal & Shute, 2012). A large diversity of medullosan ovules and pollen organs is known (*e.g*., Ramanujam, Rothwell & Stewart, 1974; Stidd, 1981; Serbet, Taylor & Taylor, 2006; Galtier, 2008). The plants occurred as minor elements in the extensive tropical forests of the Carboniferous lowland basins of Euramerica but exhibited a relatively high diversity of species and growth forms (*e.g*., Serbet, Taylor & Taylor, 2006; Dunn, 2006). The vast majority of anatomically preserved fossils originates from “coal ball” concretions of the extended Euramerican Carboniferous Coal Measures and associated clastic sediments (*e.g*., Seward, 1917; Delevoryas, 1955; Basinger, Rothwell & Stewart, 1974; Dunn, 2006). Whole-plant concepts of medullosans are rare and based on scattered knowledge of organic connections and plant architecture, derived both from anatomically preserved plant parts and compression fossils (*e.g*., Pfefferkorn et al., 1984). Restorations of Upper Pennsylvanian medullosans (*M. thompsonii*, *M. noei*) show up to 5 m high plants with straight or sinuous stems of slender stature, exhibiting large and numerous fronds (≤20) of alethopterid foliage, and adventitious roots near the stem base (Andrews, 1945; Baxter, 1949; Bertrand & Corsin, 1950; Stewart & Delevoryas, 1956). These reconstructed plants are regarded as (semi) self-supporting (Pfefferkorn et al., 1984; Wnuk & Pfefferkorn, 1984; DiMichele et al., 2006), whereas other medullosans were probably climbers, scrambler or “leaners” (Dunn et al., 2003; DiMichele et al., 2006).

In contrast to the widely investigated medullosans of the Carboniferous tropical lowland basins, medullosans from seasonally dry habitats of mainly lower Permian intramontane basins of central Europe remained poorly investigated for a long time (*e.g*., Cotta, 1832; Renault, 1875; Göppert & Stenzel, 1881; Weber & Sterzel, 1896). Thus, their growth habit, architecture, tissue functionality and palaeoecology are poorly known. Some distinct anatomical differences compared to their earlier relatives of Carboniferous lowland floras give reason to evaluate the heterogeneity of medullosan growth forms and the evolutionary mechanisms behind them (Luthardt et al., 2021).

Medullosans of Upper Pennsylvanian–lower Permian seasonally dry habitats are characterised by slender to massive stems consisting of central parenchymatous tissue, surrounded by a mostly cylindrical peripheral vascular system with considerable amounts of secondary tissues, as well as central accessory strands of variable numbers. Attached to the stem of some forms, massive petiole bases indicate that large fronds were attached, suggestively bearing alethopterid, neuropteroid and taeniopterid foliage (Luthardt et al., 2021). Five stem taxa are differentiated, encompassing *M. stellata* and varieties (Cotta, 1832), *M. porosa* and varieties (Cotta, 1832), *M. leuckartii* and varieties (Göppert & Stenzel, 1881), *M. solmsii* and varieties (Schenk, 1889), all from Chemnitz, as well as the questionable taxon *M. gigas* (Renault, 1896) described from Autun (France). Organ connections with *Myeloxylon* type petioles were first shown by Grand’Eury (1877). Moreover, evidence of further organ connections is missing, especially because the foliated apical stems are hardly preservable in most taphonomic environments.

In our contribution, we present the three-dimensionally preserved crown of a medullosan individual (*M. stellata* var. *typica* Cotta) from the Chemnitz Fossil Forest. The specimen consists of several radially arranged fronds with abundant *Alethopteris schneideri* foliage preserved as adpressions, attached to an anatomically preserved upper stem section, including the apex (Rößler et al., 2012; Rößler, 2021). Based on the fossil material, we can widely reconstruct the original anatomy and architecture of this foliated upper stem and apex. The results enlighten our understanding of Upper Pennsylvanian–lower Permian medullosans of seasonally dry habitats and represent an essential step towards new whole-plant concepts of these enigmatic ancient seed plants.

## Materials and Methods

### Geological setting and taphonomy

**Figure 1:**
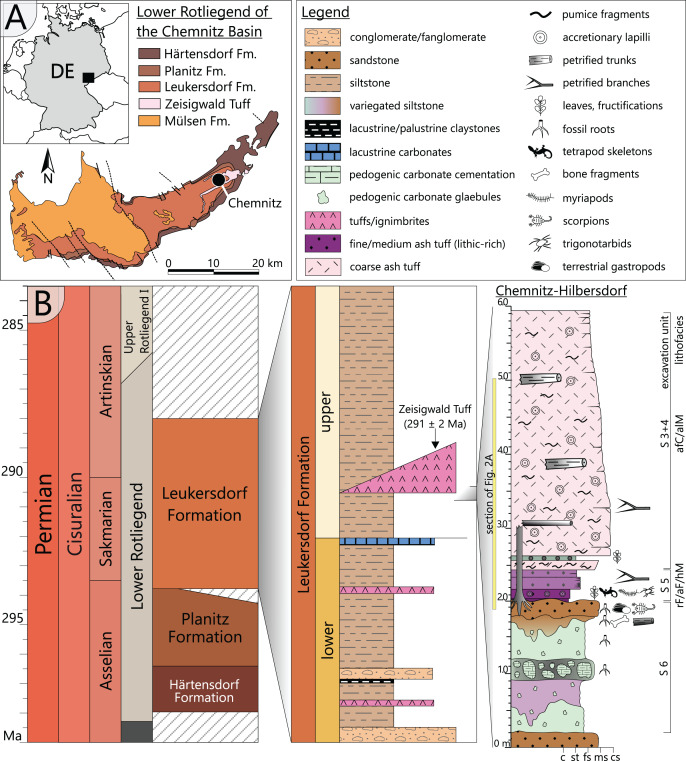
Geological setting and stratigraphy of the Chemnitz Fossil Forest. (A) Geographic position of the locality in the intramontane Chemnitz Basin (black dot). (B) Stratigraphic level of the Leukersdorf Formation, in which pyroclastics of the Zeisigwald Volcanic Complex (ZVC) are intercalated; on the right: excavation profile of the basal Zeisigwald pyroclastics exposed at the Chemnitz-Hilbersdorf excavation site comprising lithological units S 6 to S 3. Lithofacies abbreviations explained in Fig. 2 (after Luthardt et al., 2018).

The Chemnitz Fossil Forest represents an *in-situ* preserved forest ecosystem of early Permian age that depicts a narrow window in the geological past of the Chemnitz Basin (Fig. 1A). As one of the late Paleozoic intramontane basins, it developed during the post-orogenic phase of the Variscan belt in the (sub)tropical Northern Hemisphere (Schneider, Rößler & Fischer, 2012). The sedimentary succession of the Chemnitz Basin predominantly encompasses siliciclastic and volcaniclastic sediments of earliest to latest Permian age, with a significant hiatus in the middle Permian (Schneider, Rößler & Fischer, 2012). The Chemnitz Fossil Forest was found recorded in the Leukersdorf Formation (Sakmarian–Artinskian; Schneider et al., 2020), a homogenous succession of silty-sandy siliciclastics of an alluvial plain in a basin-central position (Fig. 1B). The deposits are classified as “wet red beds”, showing fluvial sedimentary patterns, immature palaeosols (predominantly calcisols) and both lacustrine and palustrine horizons (Schneider, Rößler & Fischer, 2012). The forested landscape of the Chemnitz Fossil Lagerstätte was buried by pyroclastic deposits of the Zeisigwald Volcanic Complex (ZVC). The basal pyroclastic horizons reveal a U-Pb radioisotopic age of 291 ± 2 Ma (Fig. 1B; Luthardt et al., 2018). Due to the rapid conservation by the pyroclastics, large parts of the ecosystem were preserved, encompassing remains of plants, animals and fungi that formed a complex food web and provide various insights into environment interactions (Rößler et al., 2012; Rößler, 2021; Dunlop & Rößler, 2013; Feng et al., 2014; Dunlop et al., 2016; Luthardt, Rößler & Schneider, 2017; Luthardt, Merbitz & Rößler, 2018; Spindler et al., 2018; Rößler, 2021). The palaeoclimate was sub-humid with distinct seasonal droughts (Luthardt, Rößler & Schneider, 2016). Hygro- to mesophilous elements (cordaitaleans, medullosaleans, psaroniaceous tree ferns, calamitaleans) dominated the plant community of the Chemnitz Fossil Forest. Locally, the forest thrived at wet sites, characterised by a near-surface groundwater level (Luthardt, Rößler & Schneider, 2016). Differences in substrate characteristics and the floral composition point to site-specific variations within the fossil ecosystem. At the Chemnitz-Hilbersdorf locality, different taxa of medullosaleans were growing in abundance on a colour-mottled calcic palaeosol, overlain by an immature, well-drained and well-aerated clastic topsoil (Luthardt, Rößler & Schneider, 2016; Rößler, 2021).

Floral and faunal elements of the Chemnitz Fossil Forest were rapidly buried within their habitat by pyroclastic deposits resulting from a nearby eruption of the ZVC and thus represent a significant T^0^ taphonomic assemblage *sensu stricto* (Rößler et al., 2012; Luthardt et al., 2018). Ascent of a highly fractionated, silicic and volatile-rich magma caused an explosive eruption of phreato-plinian style by coming into contact with groundwater. As a result, up to 90 m thick deposits of highly fragmented pyroclastic material accumulated in vast parts of the NE Chemnitz Basin. Minor fallout deposits at the base of the succession reflect the initial low-energy phase of the eruption, followed by major deposits of the first wet, later dry and hot pyroclastic density currents during the high-energy ignimbritic phase (Luthardt et al., 2018).

**Figure 2:**
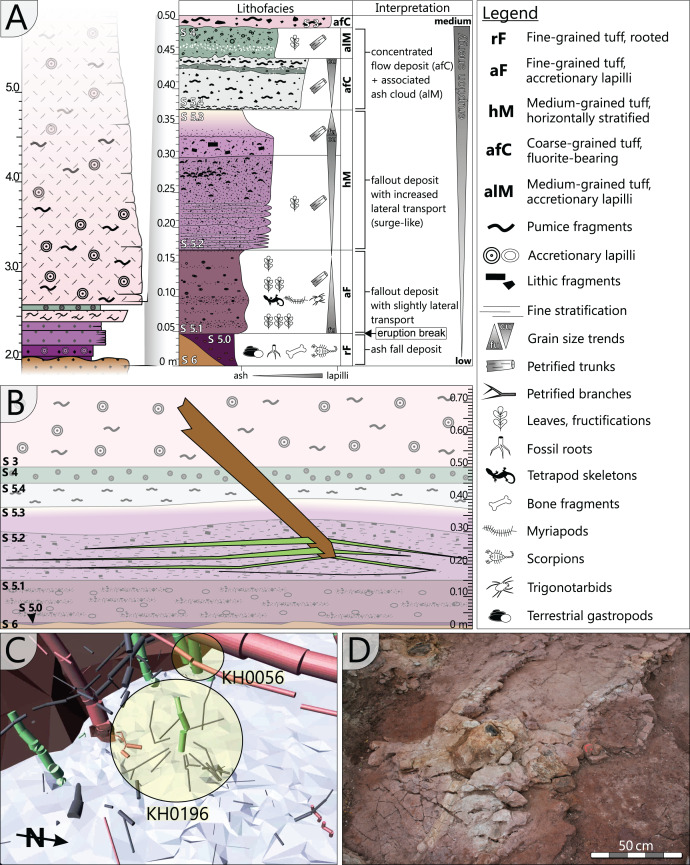
Detailled taphonomy of the upside-down embedded medullosan crown of KH0196. (A) Detailed excavation profile of Fig. 1B (right) and interpretation of the basal pyroclastics (modified from Luthardt et al., 2018). (B) Schematic vertical position of KH0196 with attached fronds; colours and numbers of pyroclastic horizons refer to those of (A). (C) 3D image of the excavation showing the position of KH0196 and its potential basal stem KH0056 (white: palaeosol surface; red trunks: calamitaleans; green trunks: medullosans; grey trunks: cordaitaleans/conifers; courtesy V. Annacker). (D) KH0196 partly uncovered during excavation work in Chemnitz-Hilbersdorf.

**Figure 3:**
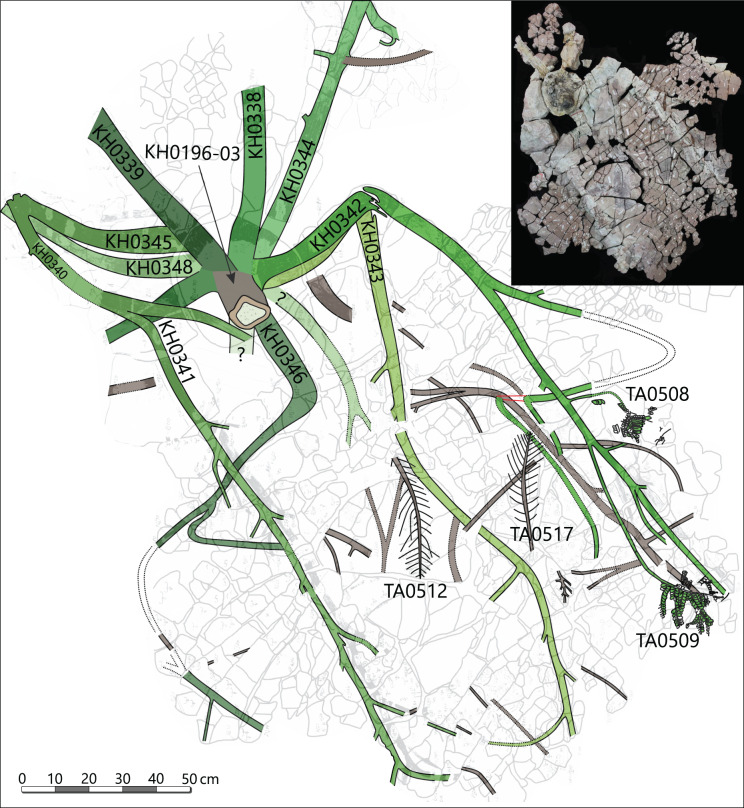
Documentation of KH0196 with ten attached fronds (KH0338-348) and some prepared foliage adpressions (TA0508, −509, −512, and −517). Records are based on preparation work of larger axes and examination of tuff fragments. Axes in brownish colour have no affiliation to one of the fronds.

The specimen was found during excavation work in Chemnitz-Hilbersdorf, in 2010. The Hilbersdorf excavation exposed pyroclastics of the initial fallout phase and the basal deposits of the ignimbritic phase (Fig. 1B). The excavation profile shows a sharp transition between the clastic palaeosol (S 6) in which *in-situ* plants are rooted and the overlying pyroclastics (S 5/4/3; lithofacies *rF*/*aF*/*hM*/*afC*/*alM* after Luthardt et al., 2018). These pyroclastics are regarded as a series of fallout and low-energy density current deposits (Fig. 2A). The KH0196 foliated stem was found embedded upside-down in the pyroclastic horizons of units S 5, S 4 and S 3 (Fig. 2B; Rößler et al., 2012). It had an oblique position with its basal stem oriented in SW direction (Figs. 2B–2D). Its uppermost 0.20 m were covered by lithofacies *afC* (S 5.4 + S 3) and *alM* (S 4). The lower-lying part with the apex and attached fronds were found directly resting on the surface of S 5.1 (*aF*) and embedded in S 5.2/5.3 (*hM*). In the area around the crown, the pyroclastics of *hM* were bulging several centimetres high, with the maximum thickness occurring around the stem (Fig. 2B). In proximity to foliage, the surrounding tuff was somewhat finer-grained compared to the composition of lithofacies *hM*. Ten mostly incomplete fronds (KH0338–KH0348) were horizontally embedded and with their basal parts radially arranged around the stem apex (Figs. 2D, 3). Their attachment to the stem is evidenced from the tuff block KH0196-03 containing the apical stem part (Fig. 3). Corresponding to their attachment position to the stem, the fronds exhibit slightly different vertical levels in the pyroclastic sediment. Some of the fronds (KH0341+345, −342, −343, −346) are bent ca. 0.50 m apart from the stem and aligned in the SW direction. From the other fronds mainly aligned to the opposite orientation (KH0338, −339, −344, −348), only the basal part up to a length of 0.80 m remained.

Alethopterid foliage was found closely related to the fronds and most abundant in between KH0341 and −342. The fragments contain numerous adpressions of pinnae and pinnules in various orientations (predominantly horizontal) and several superposed layers. Most of the pinnae are nearly entirely preserved, whereas some others are incomplete, bent or broken due to mechanical deformation. Due to the dense and sometimes apparently chaotic arrangement of pinnae, their affiliation to major rachises of the fronds is often challenging. Associated with alethopterid foliage, several detached fragments of calamitalean and walchian leafy twigs were found.

### Excavation techniques

The fossil material of the medullosan crown was recovered by the corresponding author and colleagues during excavation work at the Chemnitz-Hilbersdorf locality between 2008 and 2011 (N 50°51′10″, E 12°56′46″). Detailed documentation accompanied the excavation, capturing the three-dimensional position and orientation of the fossil plant parts in the pyroclastic deposits by tachymeter (Rößler et al., 2012). The fossil comprises several separately collected specimens with different labels. Label “KH” denominates anatomically preserved or partly silicified plant parts, whereas label “TA” indicates plant parts preserved as adpressions (see File S1). Specimen KH0196 represents the silicified stem, consisting of three major segments (KH0196-01 to -03). The ten partly silicified fronds were labelled as KH0338 to KH0348. The foliage was collected in dm-thick pyroclastic rock slabs in a wide area around the petioles at different vertical levels (*e.g*., TA0478, TA0507–515; TA0517–522, TA0524).

### Preservation and preparation work

**Figure 4:**
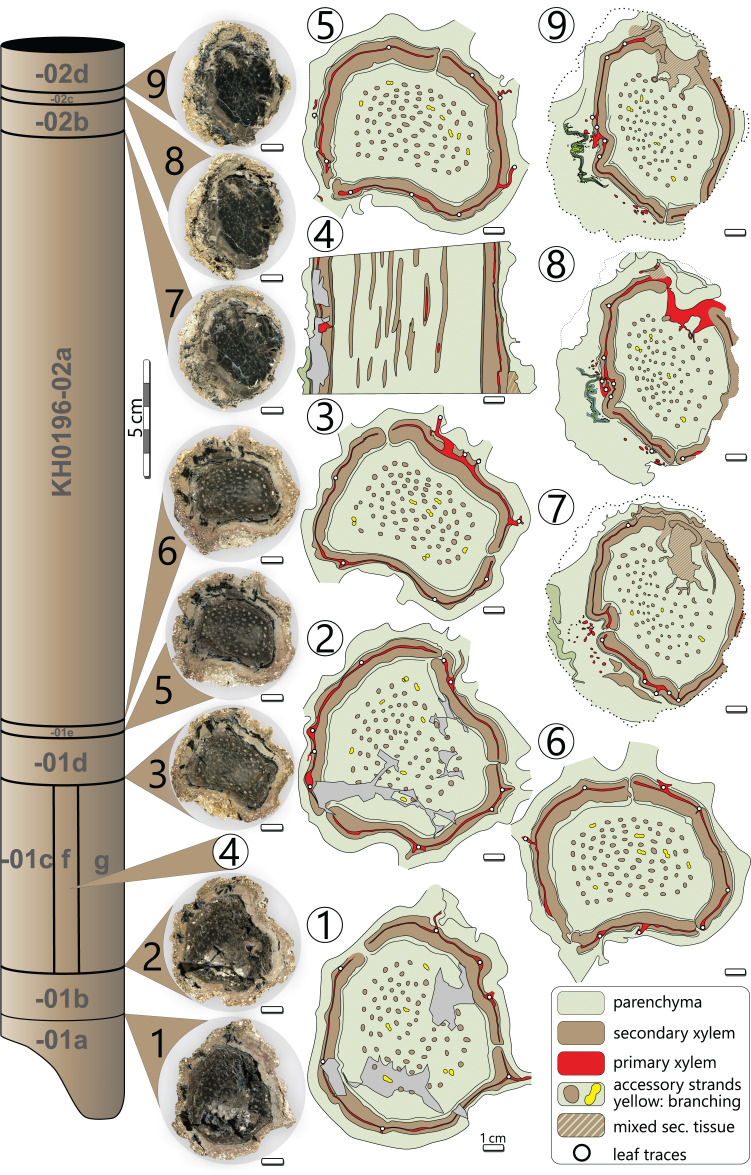
Stem anatomy of eight transverse sections and one tangential section (4) of KH0196-01 and -02. Sketches show the general arrangement of significant stem anatomical units comprising the wide central parenchymatous tissue with numerous accessory strands, the cylindrical peripheral vascular segment with secondary tissues and primary xylem, from which leaf traces emerge, and the cortex.

Processes of early diagenesis silicified the stem and thus caused its anatomical preservation. Broken parts were glued together and stabilised by cyanoacrylate and PVB. Stem segments KH0196-01 and -02 were cut into several slices so that eight polished transverse and three longitudinal sections (KH0196-01 e/f/g) from an 80 mm long segment are available for documentation of anatomical details (Fig. 4). Preservation quality of stem tissues allows for documentation of major stem anatomical features in all sections. The upper stem segment KH0196-03, representing the plant apex with attached petioles, was left in its tuff block, enabling reconstruction of the position of bordering rock slabs, which contain attached petioles and foliage adpressions.

Fronds are predominantly preserved as adpressions but are partly silicified in proximity to the stem, where petioles were thicker, *e.g*., KH0338. The latter frond segment delivered polished tangential sections. Tiny roundish cavities frequently occur within petiole sections filled by clay minerals and referred to as relicts of *in-situ* developed garnet. Common garnet formation seems to have been related to fluid-rich parenchymatous tissues (Rößler, 2021).

Foliage organs and more distal rachises left three-dimensional moulds in the tuff (adpressions), which were secondarily filled by Fe and Mn oxides. However, relics of the original organic matter are not preserved. Adpressions provide information on the morphology of the upper and lower sides of the leaves and their three-dimensional shape in transverse section. The preparation of foliage generally started from the top of rock slabs by using a compressed air-operated graver. According to their embedding in the tuff, either the lower or upper pinnule surface was uncovered.

### Image analysis techniques

Polished sections were digitised using an Epson Perfection V330 photo scanner with high resolution (1,200 × 1,200 dpi). For microscopic analysis a Nikon SMZ 1500 stereomicroscope was used, mounted with a Nikon DS-Fi 1 digital camera and NIS Elements software (version 3.2). The NIS Elements software was also used for anatomical measurements. The parenchyma portion of secondary xylem was estimated from calculating the surface percentage of rays in a radial section of KH0196-02c_I (see File S2).

A 0.25 m long stem segment (KH0196-02a) was photogrammetrically digitised to visualise frond scars left on the stem surface. For this, 22 photos covering 360° of the stem’s circumference were captured using a Panasonic Lumix DMC-G3 camera equipped with an Olympus Digital objective lens (40 mm focal width/f 5.4). The 3D model originated from using the open-source software Regard 3D.

**Figure 7:**
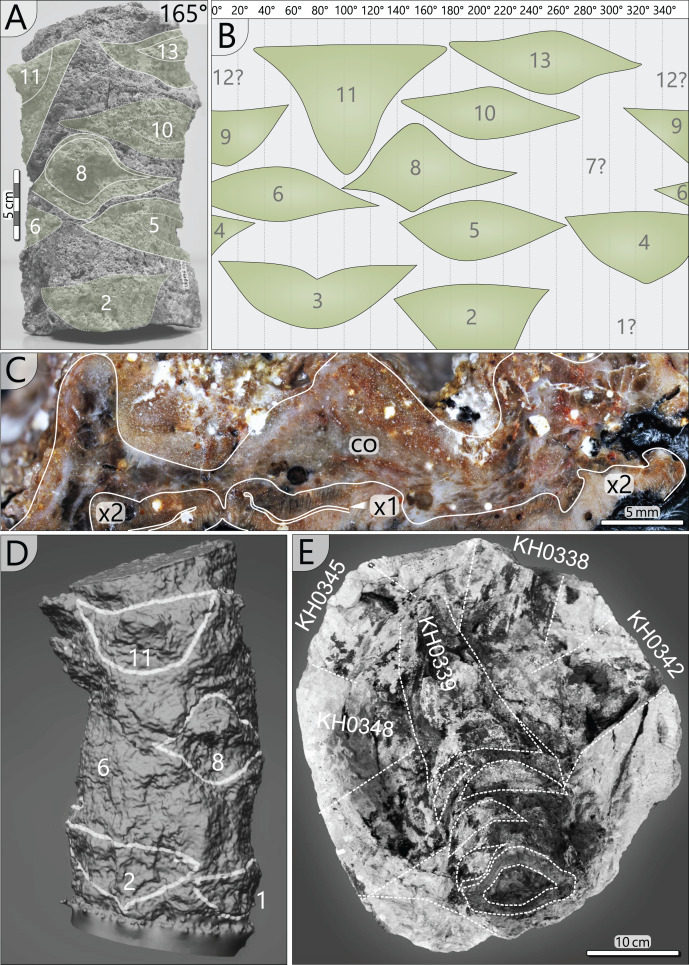
Petiole attachment from the apex (KH0196-03) and older petiole scars of stem segment KH0196-02a. (A) Stem fragment KH0196-02a with relicts of old frond scars. (B) Documentation of all frond scars displaying the enrolled stem cylinder and revealing the 3/8 phyllotaxis of frond production. (C) Old frond scar in transverse section (no. 3 in Fig. 4), probably showing poorly preserved abscission tissue, where cortex (co) is indicated (x1–primary xylem; x2–secondary xylem). (D) 3D image of stem section -02a showing the morphology of old frond scars. (E) Top view on the tuff block of KH0196-03 containing the apex of the plant and attached basal petioles. The stem shows older frond scars directly under the attached active fronds indicating abscission.

### Plant reconstruction

Stem reconstruction is based on eight transverse sections and three longitudinal sections from a 0.41 m long stem segment (KH0196-01 to -03), representing the part from the broken base up to ca. 0.20 m below the apex (Fig. 4). Transverse sections were used to measure and reconstruct thickness and architectural variations of the stem tissues, but also of single anatomical elements such as the central parenchymatous tissue, central accessory strands, wood cylinder, the primary xylem, cortex and older petiole bases attached. Arrangement of the fronds, including attachment angles, proximal diameter and distances between them, was documented from the 0.20 m long section of KH0196-02a (primary data provided in File S2).

The most completely preserved fronds attached to KH0196 are KH0341 (2.90 m long) and KH0342 (2.00 m long). Both fronds show bifurcation and attachment of secondary rachises; KH0342 also shows attached foliage (Fig. 3). KH0341 includes the specimen numbers KH0340 and −345, as frond parts were initially regarded as separate fronds. The fronds KH0343 (2.20 m) and −346 (1.05 m) also show bifurcation and are documented over a considerable length. Numerous pinnules were found closely associated with the fronds (*e.g*., TA0508 + 509). Additionally, well-preserved specimens TA0512 and −517 were taken as reference for large outer secondary pinnae. Various measurements on length, width, distances and attachment angles of pinnae and pinnules were performed and used to model the architecture of the entire frond. The length of the main rachis is estimated based on the ratio of its thinning per length, which was calculated from the preserved frond parts and is 33 mm/m. The same procedure was performed for secondary rachises. Their estimated sizes were verified by the secondary pinna of TA0509, attached to KH0342 and preserved in complete length. Primary data on frond metrics is provided in File S3. Reconstruction of missing parts of the frond is mainly based on the bilateral symmetry and repetition as important plant-architectural principles, supported by knowledge of medullosan fronds from literature.

Crown reconstruction is based on the organ connection of fronds and the stem. The arrangement of fronds is reconstructed from excavation documentation and older petiole scars at the stem surface.

**Systematic palaeontology**

Class Pteridospermopsida

Order Medullosales

Family Medullosaceae

Genus *Medullosa* Cotta (1832)

Species *Medullosa stellata* Cotta (1832)

**Emended diagnosis.** long and slender stem of infinite diameter, sometimes with periodic thickenings; peripheral stem vascular segment with a concentric zone of primary tissues surrounded by centripetally and centrifugally grown secondary xylem and phloem; inner wood normally thicker than outer wood; peripheral vascular segment shows irregular interruptions in cross-section; leaf traces emerge from primary tissue of the peripheral vascular segment; wide central parenchymatous tissue with a variable number of accessory strands showing primary tracheids and radially arranged secondary xylem and phloem; thin parenchymatous cortex showing thickening where petiole bases were attached. Diagnosis emended from Cotta (1832, p. 65) and Weber & Sterzel (1896).

**Figure 5:**
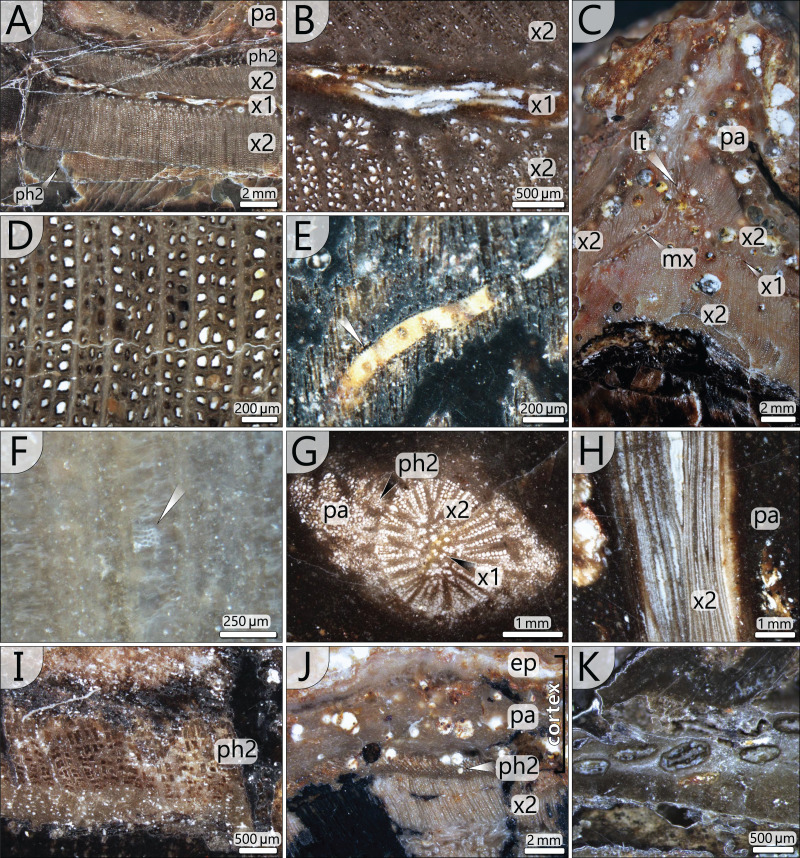
Stem anatomical details at microscopic scale. (A) Part of peripheral vascular segment and its primary and secondary tissues (section 7); (B) Close-up of (A) showing horizontal and vertical meta-xylem tracheids. (C) Inactive leaf trace of an abscised frond showing horizontal metaxylem tracheids in the primary xylem, migrating out of the vascular segment (section 1). (D) Secondary xylem structure of the peripheral vascular segment (section 8). Note the thick walls and comparably thin parenchymatous rays. (E) Metaxylem tracheid with serrate wall thickenings (white arrow, section 5). (F) Faintly preserved wall structure of secondary xylem tracheid showing relict details of multiseriate pitting (section 4). (G) Central accessory strand surrounded by parenchyma (section 5). (H) Central accessory strand in longitudinal section (section 4). (I) Detail of secondary phloem occurring in the periphery of the peripheral vascular segment (section 3). (J) Stem cortex structure composed of parenchymatous layer and a supposed epidermis (section 5). (K) Flattened secretory canals in transverse section close to vascular element shown in Fig. 5C (section 8). abbreviations: ep, supposed epidermis; lt, leaf trace; mx, metaxylem tracheids; pa, parenchyma; x1, primary xylem; x2, secondary xylem; ph2, secondary phloem.

**Figure 6:**
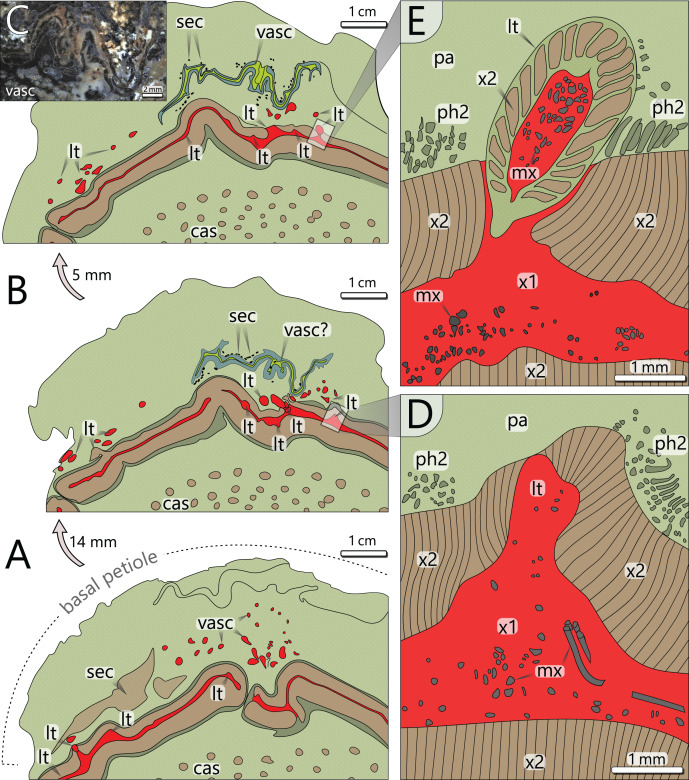
Architecture of the vascular system at three levels of an attached petiole (sections 7–9). See Fig. 4 for colour code. (A) Petiole base indicated by thickened cortex; peripheral vascular segment is bulging and shows a typical gap; leaf traces emerge from the primary xylem, and branch inside the cortex parenchyma. (B) Vascular elements in the cortex of (A) are replaced by a birdwing-shaped multi-layered structure (single vascular element?) with several secretory canals at its margins; the gap of the peripheral vascular segment is closed, but several leaf traces still emerge. (C) Uppermost section 9 shows continuous leaf trace emission from the peripheral vascular segment and the supposed birdwing-shaped vascular element. (D) Close-up of emerging leaf trace of section 8 in (B) associated with horizontal metaxylem tracheids migrating towards the leaf trace. (E) Same leaf trace as in (D), but now completely developed and surrounded by secondary xylem. abbreviations: cas, central accessory strands; lt, leaf trace; mx, metaxylem tracheids; pa, parenchyma (cortex); ph2, secondary phloem; sec, secretory canals; vasc, vascular element(s); x1, primary xylem; x2, secondary xylem.

**Lectotype.** The specimen K3004 (Museum für Naturkunde Chemnitz), selected by Luthardt et al. (2021). Syntypes are MB.Pb 2011/0950 ((Cotta, 1832, Pl. XIII, Figs. 4–6) and MB.Pb 2021/0877 Pl. XIII, Fig. 1), stored at palaeobotanical collection (Museum für Naturkunde Berlin).

**Type locality.** Chemnitz Fossil Forest, Chemnitz-Hilbersdorf.

**Stratigraphy.** Zeisigwald Volcanic Complex, Leukersdorf Formation; Age: 291 ± 2 Ma, early Permian (Luthardt et al., 2018).

**Description (KH0196)**

The silicified stem section is 0.56 m long and 95–130 mm (⌀ 105 mm) in diameter. Its diameter slightly decreases from base to top by ca. 20 mm. In transverse sections, the stem is roundish to oval and shows concave embayments (*e.g*., sections 3, 5, 6 in Fig. 4), where petioles were attached. Major stem anatomical elements are the central parenchymatous tissue with numerous accessory strands, a cylindrical peripheral vascular segment from which leaf traces emit, and a parenchymatous cortex of variable thickness. A periderm is absent.

*Central parenchymatous tissue and accessory strands*: The mean diameter of the central parenchymatous tissue decreases from the base (84 mm) to the top (62 mm). The parenchyma consists of isodiametric, thin-walled cells. Embedded in the parenchyma, accessory strands are built up of primary xylem in the centre and secondary xylem and phloem in the periphery (Figs. 5G, 5H). In the primary xylem, metaxylem tracheids are recognised. The secondary xylem is composed of radial tracheid rows and medullary rays. In the transverse section, tracheid lumina are 7–92 µm wide (⌀ 37 µm; *n* = 123), and thus somewhat smaller compared to the tracheids of the peripheral vascular segment). Secondary phloem occurs subsequent to the tracheid rows and is usually a few cells wide. It indicates the presence of a bifacial cambium around each of the accessory strands.

The accessory strands have a mean diameter of 2.3 × 1.7 mm in the basal section KH0196-01. Towards the stem apex, the mean diameter distinctly decreases to 1.7 × 1.2 mm, in section KH0196-02. Their number at all transverse sections is constantly high but slightly varies between 77 and 87 (Fig. 4). The strands are bisecting and anastomosing, and thus forming a cylindrical network in the stem centre. Distance between adjacent strands is more or less constant, leading to a sub-symmetrical arrangement in transverse sections, which appears circular or spiral. The peripheral strands exhibit a minimum distance of 10 mm to the inner margin of the peripheral vascular segment. At the level of sections 7, 8 and 9 (Fig. 4), the accessory strands show an exceptional structural anomaly (top, right). Six to ten of the peripheral strands are merged to form a structure of hardly identifiable tissue that breaks through the peripheral vascular segment and migrates towards the stem periphery, most probably to form a stem-borne plant organ of unknown affinity. Its basal part is triangular in the transverse section, comparable to petiole bases, but is distinctly smaller (<30 mm).

*Peripheral vascular segment*: In transverse section, the peripheral vascular segment represents a subcircular ring of primary and secondary tissues around the central parenchymatous tissue. It is on average 11 mm wide in the basal section, decreasing to 7 mm in the upper sections. Its three-dimensional shape may be best described as a hollow cylinder. At the level of petiole scars, the segment is occasionally bulging and regularly shows an oval opening (Fig. 6A).

The primary xylem occurs in a narrow zone between the inner and outer secondary xylem (Fig. 5A). It consists of xylem parenchyma and horizontally and vertically oriented metaxylem tracheids (Figs. 5B, 5E). Metaxylem tracheids are up to 250 µm in diameter, thus distinctly larger than secondary tracheids, and show serrate secondary wall thickenings (Fig. 5E). Due to preservation bias, protoxylem and primary phloem were not identified. At the exocentric periphery of the primary xylem, leaf traces are emerging (see below).

The peripheral vascular segment exhibits an endocentric and an exocentric zone of secondary xylem and phloem around the primary xylem (Fig. 5A). Secondary growth occurred centripetally and centrifugally from one bifacial cambium, which is indicated by a few millimetres thin zone of secondary phloem occurring both at the outer and the inner margin of the secondary xylem (Figs. 5A, 5I). The centripetal secondary growth was dominating, resulting in a slightly wider endocentric wood zone. Tracheids of the secondary xylem are arranged in 1–4 rows in between thin parenchymatous rays (Fig. 5D). The overall parenchyma percentage is low (ca. 12%). Tracheids are radially elongated and exhibit wide, oval lumina with radial diameters of 32–113 µm (⌀ 73 µm) and tangential diameters of 7–68 µm (⌀ 38 µm; *n* = 197). Secondary cell walls are thick compared to the lumina, with 48 µm on average. Preservation allows in part the distinction of primary and secondary walls (Fig. 5D). Pitting structures are poorly preserved, but multiseriate pitting is suggested from Fig. 5F.

*Leaf traces*: In each of the investigated transverse sections, 5–9 leaf traces were documented (Fig. 4). They are <5 mm wide and originate from the exocentric periphery of the primary tissue of the peripheral vascular segment, which is indicated by an outward bulging of the primary xylem zone (Figs. 6D, 6E). Some leaf traces reveal a clear connection with horizontal metaxylem tracheids of the primary xylem (Figs. 5C, 6D). Initial leaf traces grew horizontally or obliquely upward toward the stem periphery and may possess secondary xylem around the primary tissue (Fig. 6E). After their emergence, leaf traces are dissected and migrate towards the petiole base (Figs. 6A–6C). Their organisation at the petiole bases is complex. In sections 7–9 (Fig. 4), a petiole base is documented at three vertical levels (Figs. 6A–6C). At every level, leaf traces emerge from the primary xylem zone; altogether at least five were counted. In basal section 7 (Fig. 6A), the vascular strands in the centre of the petiole base seem to form a ring-like pattern, whereas the peripheral vascular segment is distinctly bulging to the inside. In sections 8 and 9, most vascular strands are obviously merged to a single bird-wing-shaped vascular element, which exhibits a multi-layered internal setup (Figs. 6B, 6C). Many tiny oval tubes identified as secretory canals occur in proximity to this element (Fig. 5K).

*Cortex*: The cortex is represented by a parenchymatous tissue surrounding the whole stem cylinder. It varies in thickness (4–7 mm) and probably causes an irregular morphology of the stem surface (Fig. 4). Internally, the cortex is composed of tangentially elongated parenchyma cells and surrounded by a thin layer of undifferentiated cells, probably representing an epidermis layer (Fig. 5J). At the petiole bases, the cortex significantly thickens (Figs. 6A, 6B). No evidence of a stem periderm was found, which might be due to preservation bias.

*Petiole attachment*: The arrangement of fronds can be reconstructed from remaining scars at the outer stem surface and from the fronds that are still attached. In a 0.23 m long stem segment (KH0196-02a), thirteen petiole scars were documented (Figs. 7A, 7B, 7D). Scars are helically and densely arranged in a clockwise rotation towards the top, indicating orthotropic growth of the fronds. A single petiole base extends over almost one-third of the stem circumference. The resulting angle between two consecutive petioles varies between 110° and 150°, and the average vertical distance between them is 18 mm. The angles between the attached fronds and older petiole scars indicate a 3/8 phyllotaxis corresponding to the Fibonacci sequence, implicating a mean angle of around 135° between two consecutive fronds. Older frond scars typically show a thickening in the middle of the scar but sometimes also relict leaf traces (Figs. 7C, 7D). Preservation of the cortex in Fig. 7C is poor and obscures differentiation of a potential sclerenchymatous abscission tissue.

The tuff block of KH0196-03 contains the silicified apex of the medullosan stem with the helically attached fronds, which are preserved in the surrounding tuff (Fig. 7E). Their ontogenetic sequence remains unclear. Frond scars are visible on the stem surface, suggesting abscission of older inactive fronds (Fig. 7E).

Class Pteridospermopsida

Order Medullosales

Family Medullosaceae

Genus *Alethopteris* Sternberg, 1825 emend. Zodrow & Cleal, 1998

Species *Alethopteris schneideri* Sterzel (1881) Barthel (1976a)

**Selected synonymy.** *Callipteridium schneideri* sp. nov.–Sterzel (1881, p. 262).

*Callipteris weberi* Sterzel–Weber & Sterzel (1896, p. 111).

*Alethopteris grandini* Brgt.–Franke in Potonié (1912, pro parte, p. 168, Fig. 1).

*Alethopteris grandini* Brgt.–Remy & Remy (1959, p. 166, Fig. 141 a, b)

*Callipteris weberi* Sterzel–Sterzel (1918, p. 286–288, Pl. 8, Fig. 87, Pl. 9, Fig. 88, 89, Pl. 15, Fig. 88a).

*Alethopteris Schneideri* Sterzel nov. comb.–Sterzel (1918, p. 289–292, Pl. 9, Fig. 93, Pl. 10, Fig. 92, 94, Pl. 15, Fig. 93a).

**Emended diagnosis.** Bifurcated, tripinnate fronds, more than 3.2 m long (adult plant); transition of tertiary pinnae to higher-order elongated tertiary pinnules at the apices of secondary pinnae; pinnules morphologically variable, but mostly elongated to short linguiform with obtuse apex, ca. 8–15 mm long; obliquely inserted to pinnule axis; pinnule base basiscopically extending and acroscopically deeply incised (usually to the midvein); long and straight prominent midvein extending up to 3/4 of pinnule length; dense venation, mostly double forked lateral veins, which are acutely attached to midvein, rapidly bending and reaching almost perpendicularly the pinnule margin. Diagnosis emended from Barthel (2006).

**Lectotype.** Barthel (1976b) selected the specimen figured by Sterzel (1918, Taf. 10, Fig. 94), stored in the collection of the Geological Survey of Saxony, Freiberg (spec. number RS2018-383).

**Type locality**. Mine shaft “Deutschland-Schacht” (501 m depth), Oelsnitz, Chemnitz Basin.

**Stratigraphy.** *Alethopteris schneideri* occurs in Gzhelian–Sakmarian strata (Upper Pennsylvanian–lower Permian). In many European basins, *A. schneideri* is a significant species for the lower Permian Rotliegend stratigraphic interval. The lectotype is of Asselian age (lowermost Permian, Härtensdorf Fm.).

**Description**

**Figure 8:**
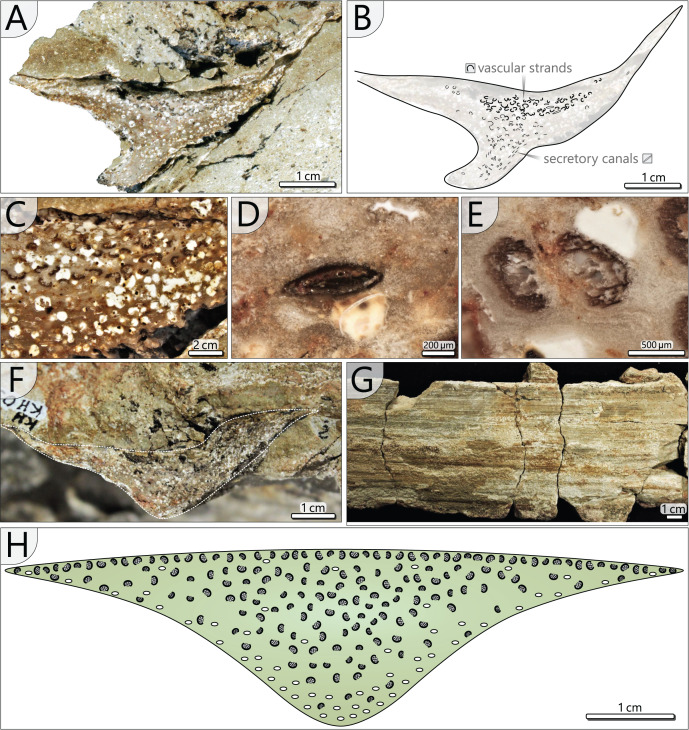
Petiole anatomy of *Myeloxylon* type fronds attached to KH0196. (A) Transverse section of anatomically preserved, but taphonomically deformed part of KH0338. (B) Sketch of the section in (A) showing vascular strands and secretory canals. (C) Densely arranged vascular segments in KH0338 (white roundish aggregates represent kaolinitic pseudomorphoses of former garnet, which has grown in the fresh plant tissue shortly after burial by volcanic ash). (D) Close-up of flattened secretory canal. (E) Branching vacular strand with metaxylem tracheids partly surrounded by dark sclerenchyma plates. (F) Less deformed part of KH0338. (G) Petiole surface of the frond KH0339 showing parallel striation of sclerenchyma plates. (H) Reconstructed transverse section of a *Myeloxylon* type petiole with triangular shape, vascular segments concentrated in the centre and the top, and secretory canals predominantly occurring at the lower margins of the transverse section.

*Petiole anatomy (KH0338)*: Basal petiole transverse sections are rhomboidal and measure 90–120 mm in width and 35–85 mm in height (Figs. 7A, 7B). From their base to the level of bifurcation, petioles become more slender and triangular (Fig. 8H). After bifurcation, the shape successively changes to elliptical in the distalmost section. Anatomical details have been studied in the proximal petiole of KH0338 (Fig. 8). In the transverse section, the petiole has a flat triangular shape. It consists of parenchymatous ground tissue, in which 220–600 µm wide leaf vascular strands are abundant in the central and upper part (Figs. 8A–8C). They are recognised by their c-shaped appearance, which results from sclerenchymatous tissue partially surrounding the metaxylem tracheids (Fig. 8E). However, some strands have no sclerenchyma. Branching of the vascular strands occasionally occurs (Fig. 8E). Compressed tubes of simple architecture and diameters of 300–650 µm are recognised as secretory canals and are most abundant in the basal central region, but they also occur at the margins of the petiole (Figs. 8B, 8D). The surface of most petioles typically shows parallel striation resulting from sclerenchyma plates (Fig. 8G).

*Petiole and primary pinnae (KH0341 + 342)*: The frond of KH0341 is preserved over a length of 2.90 m (Fig. 3). It is bent at 0.67 m from its base, at an angle of nearly 180°. The petiole measures 55–80 mm in diameter and bifurcates at a distance of 1.23 m and a 25° angle. One of the two primary pinnae was documented at a length of 1.57 m but lacking the most distal part. This primary pinna is basally 40 mm wide, distally decreasing down to 14 mm. It shows opposite alternating attachment positions of secondary pinnae, six at each side with distances of 0.16–0.34 m between successive secondary pinnae (Fig. 3). The most distal part of the primary rachis is not preserved but might be several decimetres long, referred from the distal rachis diameter of 14 mm.

**Figure 9:**
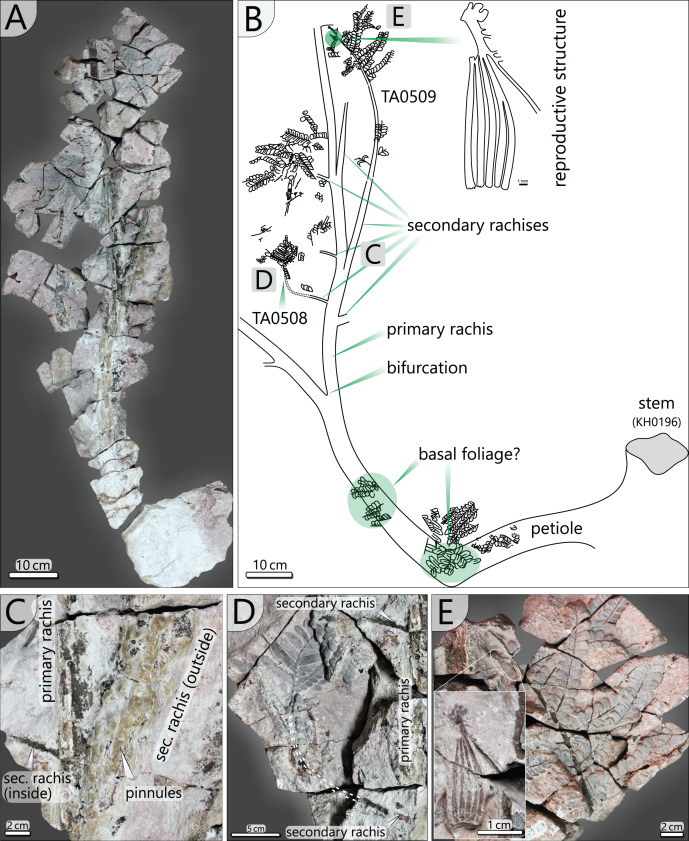
Documentation of the frond morphology (KH0342). (A) Preserved and prepared parts of frond KH0342. (B) Documentation of KH0342 with attached secondary pinnae of TA0508 and TA0509. (C) Detail of attachment of secondary pinnae to the primary rachis. (D) Partly preserved, smaller inner secondary pinna of TA0508. (E) Detail of TA0509 distal part with attached male reproductive structure at the position of a tertiary pinna.

KH0342 has a length of 2.00 m, whereas its distal part is also missing. It is bent 0.44 m distant from the stem, changing its orientation by ca. 90° (Figs. 9A and 9B). The diameter of the basal petiole is 75 mm, waning to 50 mm short before bifurcating into two primary rachises. Bifurcation occurs 0.93 m distant from the stem at an angle of 45° between both primary rachises. From both primary rachises, one is preserved over a distance of 1.12 m, but the apical part is missing. Its diameter is 35 mm directly after bifurcation, decreasing down to 20 mm at its end. Alternating basal attachments of at least three secondary pinnae (*e.g*., TA0508, 509) occur at both sides of the main rachis, showing distances of 0.25–0.38 m at the outer side of the main rachis (related to the whole frond) and 0.17–0.21 m to the inner side.

In addition, small secondary pinnae were also found closely related to the basal petioles, *e.g.*, of KH0342 (Fig. 9B). Petioles of other fronds show basal attachments of secondary rachises, suggesting foliation of basal petioles with small secondary pinnae (*e.g*., KH0341, 344).

*Secondary pinnae (TA0508*, *509*, *512, 517)*: Secondary rachises are attached to main rachises at angles in a range of 70–75°. TA0509 represents the basal of the three outer secondary rachises of KH0342 and is preserved over its whole length showing some tertiary pinnae, pinnules and a reproductive structure attached at a distal position (Figs. 9B, 9E). It departs from the main rachis at a distance of 0.38 m from the bifurcation level and at an angle of only 20°, most likely reflecting taphonomically induced bending (Fig. 9C). Its rachis is initially ca. 20 mm wide and altogether 0.72 m long. Secondary rachises oriented to the inside of KH0342 show initial diameters of ca. 6 mm. The basal, TA0508, is attached 0.24 m from the bifurcation and preserved at its original length of 0.25 m and shows attached tertiary pinnae and pinnules (Fig. 9D).

**Figure 10:**
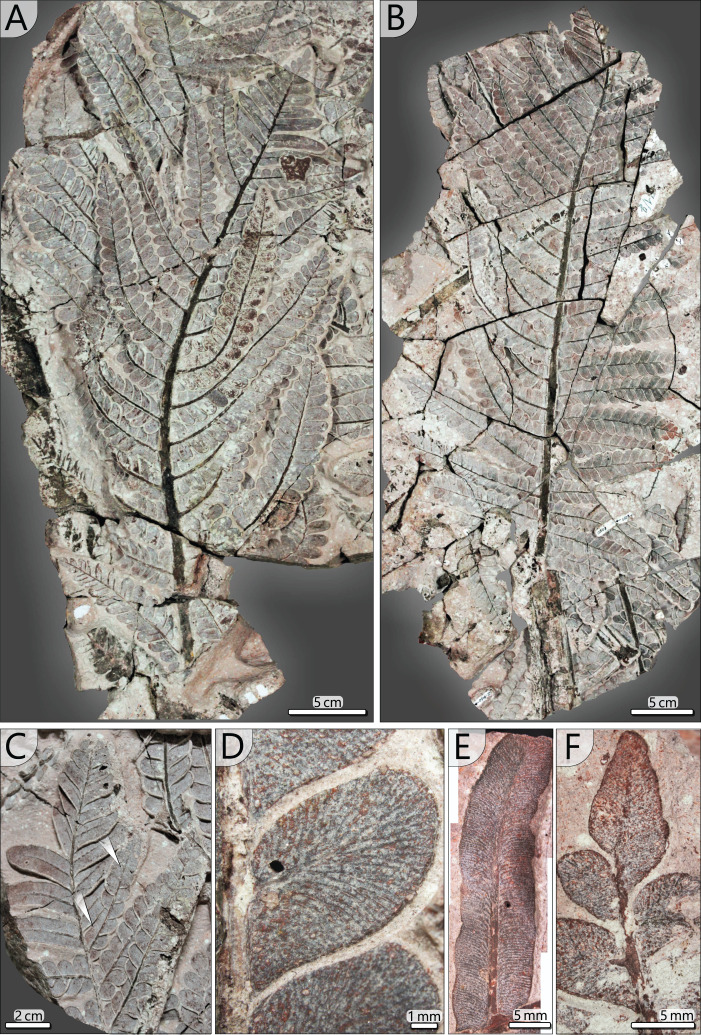
Documentation of secondary and tertiary pinnae of *Alethopteris schneideri*. (A) Outer secondary pinna of TA0512 showing transition of tertiary pinna to pinnules at the distal part. (B) Outer secondary pinna of TA0517. (C) Detail of transition from tertiary pinnae to elongated pinnules (TA0512). Note the amalgamation of pinnules in one tertiary pinna (white arrows). (D) Pinnules attached to a tertiary rachis showing details of venation patterns characteristic for *A. schneideri* (TA0508). (E) Elongated tertiary pinnule attached to the distal part of a secondary rachis showing details of venation and curved pinnule margins of suggestive segmentation into lower-order pinnules (TA0508). (F) Terminal pinnule of a tertiary pinna (TA0508).

Other large secondary pinnae are TA0512 and 517 (Figs. 10A, 10B). Both show no affiliation to one of the fronds but are nearly completely preserved and provide useful insights into pinna morphology. TA0512 is preserved over a length of 0.44 m and is estimated to be originally up to 0.35 m wide. Its main rachis is basally 8 mm in diameter. Tertiary pinnae attached to the main rachis are densely arranged. At the distal parts of secondary pinnae, tertiary pinnae are replaced by distinctly smaller tertiary pinnules and a terminal apical leaf (Figs. 10C, 10E, 10F). As attached to TA0512, twelve tertiary pinnae and ten tertiary pinnules were counted on each side of the main rachis. TA0517 is similar in shape to TA0512 and preserved over a length of 0.52 m and a maximum width of around 0.30 m (Fig. 10B). The basal main rachis measures 9 mm in diameter. Fourteen attached pinnae and ten pinnules are documented on each side; however, the apical part of TA0517 is missing.

**Figure 11:**
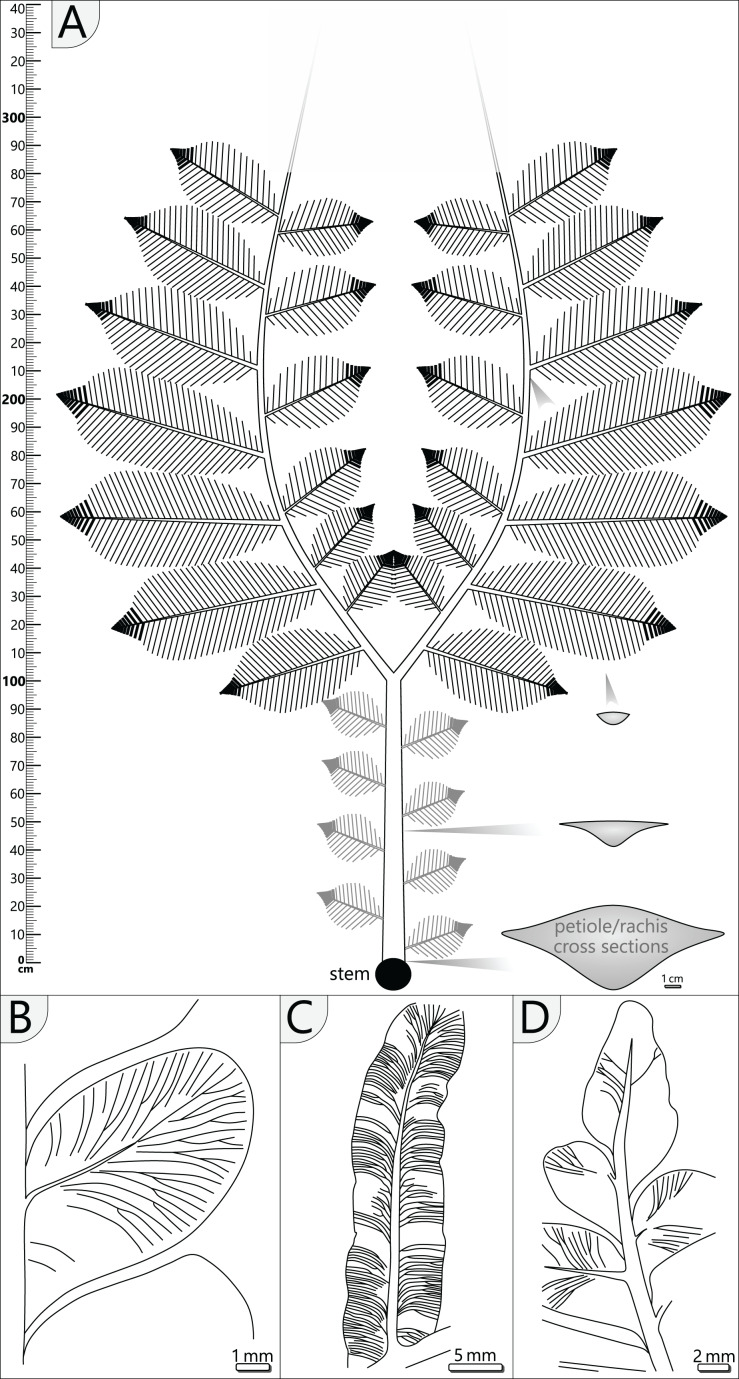
Schematic reconstruction of adult *A. schneideri* fronds attached to the *M. stellata* var. *typica* stem of KH0196. (A) Reconstructed frond with more than three metres length; unclear sections are marked in grey colour; thick lines at terminal secondary rachises indicate tertiary pinnules (see (C)). (B) Drawing of pinnule attached to tertiary rachis showing venation patterns characteristic of *A. schneideri*. (C) Drawing of tertiary pinnule attached to distal part of a secondary rachis. (D) Drawing of terminal pinnule at the tip of a tertiary pinna.

*Tertiary pinnae and pinnules (TA0508*, *509, 512, 517)*: Tertiary pinnae and pinnules are distichous and opposite with slight offsets. They are attached by a mean angle of 65° to the secondary rachis and a distance of 4–53 mm (av. 20 mm) lies between the rachises of adjacent pinnae. Consequently, tertiary pinnae are bordering directly to their neighbours (Figs. 10A, 10B). They are up to 170 mm long, 35 mm wide and bear between 10 and 19 pinnules on each side. In the distal position of secondary rachises, tertiary pinnae and pinnules rapidly decrease in size. Tertiary pinnules are strap-shaped and bear a prominent midvein, from which side veins depart at a low angle (Figs. 10E, 11C). In the further course, lateral veins curve and approach the pinnule margin at an angle of nearly 90°. Lateral veins branch basally and often branch a second time near the leaf margin. In the transition from pinnae to pinnules, sometimes intermediates occur, showing mixed morphologies of both tertiary organs (Fig. 10C).

*Pinnules*: Pinnules are 12–19 mm long and 7–9 mm wide (Figs. 10D, 11B). The midvein of pinnules is attached to the tertiary rachis by a mean angle of 65° (*n* = 17), possessing a mean distance of 8 mm (*n* = 20) to the adjacent pinnules. Opposite pinnules are offset. A tertiary pinna terminates with an apical pinnule with a sharp peak and slightly undulating margins (Figs. 10F, 11D). In general, pinnules are remarkably uniform in shape and size. They are nearly linguiform with a roundish peak (Fig. 11B). At their bases, they are acroscopically incised and basiscopically gradually descending. The central midvein is prominent in the basal half of the pinnule. Side veins depart from the midvein by low angles and immediately bend to terminate at an angle of nearly 90° at the pinnule margin. Veins are densely arranged and bifurcate once or twice after one-third of the distance towards the margin. In the transverse section, pinnules are slightly convex towards the top and enrolled somewhat at their margins, whereas the midvein represents the deepest point.

*Reproductive organ (TA0509)*: A potential reproductive structure is attached to the terminal part of the secondary pinna of KH0342 (TA0509) in place of a tertiary pinnule (Fig. 9B). The specimen represents an 18.4 mm long and 6.7 mm wide elliptical structure with five potential sporophyll tubes, each up to 1 mm wide (Figs. 9E). Other tubes might be hidden in the tuff. The visible tubes are loosely arranged but are all attached to the top of the organ. The tips of the tubes appear slightly serrated. The axis, where the organ is attached, exhibits additional basal attachments, probably once bearing similar reproductive elements.

## Discussion

### Taphonomic model

Three-dimensional preservation of nearly complete individuals is exceptionally rare in the plant fossil record. This preservation mode requires rapid and non-destructive burial of the organisms by sediment, predominantly occurring during the deposition of pyroclastics (*e.g*., DiMichele & Falcon-Lang, 2011; Opluštil et al., 2014; Trümper et al., 2021). In the Chemnitz fossil assemblage, basal deposits of the initial eruption phase of the Zeisigwald Volcanic Complex have favoured preservation of foliage adpressions in fine- to medium-grained tuff on the one side, and anatomical preservation of stems and trunks by rapid silicification after deposition, on the other (Rößler et al., 2012; Trümper, Rößler & Götze, 2018; Rößler, 2021).

Interestingly, only leaves and smaller branches were found in the tuff horizon S 5.2 (*e.g*., Luthardt et al., 2018; Rößler, 2021), distinctly contrasting the large size of KH0196. The circumstances under which KH0196 was embedded, were controlled by the mode of pyroclastic deposition and influenced by its crown architectural and stem anatomical peculiarities. The taphonomic chronology started at an early depositional phase with the initial pyroclastic fallout represented by deposits of horizon S 5.1. The wet falling ash must have covered the leaves and branches of forest trees by a thick layer. Considering ash density (2,350–3,300 kg/m^3^; Wilson et al., 2012) and the reconstructed high leaf surface area of KH0196 (ten bifurcating fronds, each up to 3.5 m long and with large secondary pinnae), the load weight of ash must have been significant, probably several hundreds of kilograms. Relicts of the load can be seen in the finer-grained tuff directly surrounding foliage, which is more similar to S 5.1, thus contrasting the coarser-grained and lithic-rich ash tuff of S 5.2. The overload may have first led to bending down and even kinking of the fronds at a critical length of around 0.5 m distant to the stem, as documented in Fig. 3. Compared to frond biomass, the stem of KH0196 is relatively thin in diameter and exhibits a high parenchyma portion, which decreases its flexuosity. In combination, high ash overload, probably accompanied by wind, and the low flexuosity of the thin apical stem may have caused its early breakage directly after deposition of S 5.1, whereas other trees in the neighbourhood remained more or less intact, at this point of the pyroclastic deposition. It is supposed that the crown fell on top of S 5.1 (Fig. 2B) without being transported over a certain distance. Therefore, the basal stem of this plant might be standing close to KH0196. One candidate is specimen KH0056, an up to 0.21 m thick stem of *M. stellata* var. *typica* affinity, which was found less than 3 m distant from KH0196, still anchored in the palaeosol and broken at the height of 2.1 m (Fig. 2C). The position of KH0196 to KH0056 would indicate that the stem of the plant was bending like trees experiencing snow overload before it broke apart and fell on the ground.

After the burial of KH0196 by fallout deposits of S 5.2, more massive stem and petiole parts were (partly) silicified, whereas more delicate organs such as rachises and foliage were replaced by inorganic iron and manganese oxides, but leaving adpressions in the adhering tuff.

### Plant reconstruction

The stem of KH0196 is of slender and regular stature and represents the upper juvenile part of a *M. stellata* var. *typica* (Weber & Sterzel, 1896). Over its whole length, the stem of KH0196 shows only minor thickening (~1 cm) from top to base (Fig. 4). The original length of the stem remains unknown. However, other stems of comparable diameters from the MfNC collection exhibit dimensions of several metres in length, which might be easily assumed for KH0196. The potential base of KH0196 (KH0056) is preserved over an upright length of 2.1 m, resulting in a combined stem length of ca. 2.5 m.

**Figure 12:**
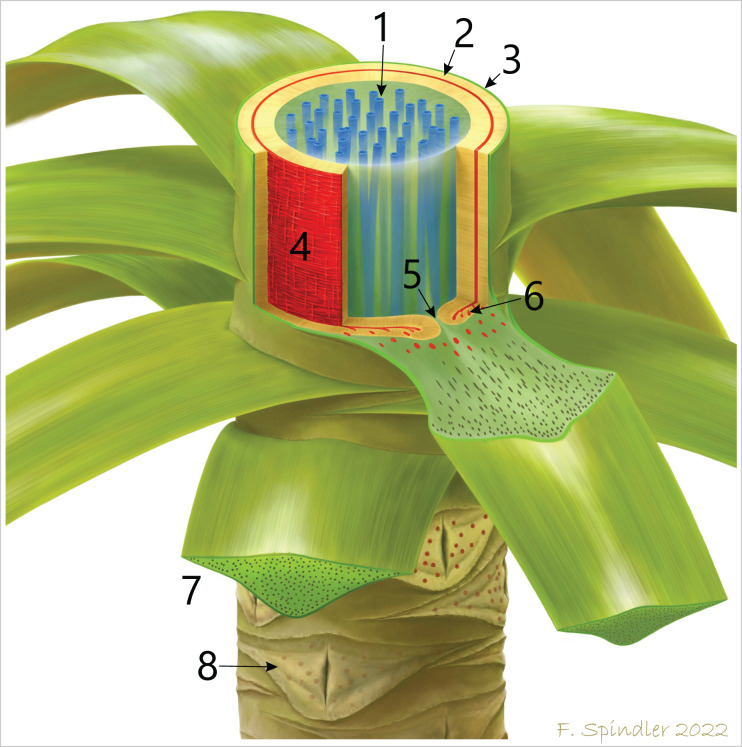
Schematic reconstruction of the stem anatomy and vascular system of *M. stellata* var. *typica* (KH0196). 1–central accessory strands (blue); 2–centrifugal and centripetal secondary xylem of the peripheral vascular segment (beige); 3–stem cortex; 4–primary vascular tissue (red) with horizontal and vertical metaxylem tracheids; 5–central furrow in attachment position of a frond; 6–leaf traces emerging from the primary vascular system and migrating into the fronds. 7–basal petiole with scattered vascular strands; 8–older leaf scars of detached fronds showing the characteristic elliptical opening in the xylem cylinder.

The organisation of the stem vascular system of *M. stellata* var. *typica* is complex and mainly characterised by the peripheral vascular segment, from which leaf traces emerge, as well as the branching and anastomosing central accessory strands, from which no leaf traces emerge (Fig. 12). The connection of accessory strands to the water-conducting system of the plant remains unknown but is expected to be visible in the obscured stem apex. Nevertheless, we cannot exclude some unknown side organs connected to the accessory strands (Fig. 4, sections 7–9). Fronds were connected by many leaf traces to the stem vascular system, emerging from the primary xylem of the peripheral vascular segment (Figs. 6, 12). Shortly after entering the stem cortex, leaf traces seem to merge to a single birdwing-shaped vascular element, from which they most likely split into numerous vascular strands in the petiole (Figs. 6B, 6C). A similar structure in *M. stellata* var. *typica* is shown in Cotta (1832; Pl. XIII, Fig. 1) and Weber & Sterzel (1896; Text-Fig. 7). However, further material needs to be studied to verify this observation.

The ten fronds found around KH0196 were helically attached to the uppermost 10–15 cm of the stem, following a 3/8 phyllotaxis (Figs. 3, 7). Below, densely arranged petiole scars at the stem surface indicate that older fronds were abscised when they became inactive or died off, even though an abscission layer is not clearly distinguishable (Fig. 7C). We assume that most of the ten attached fronds were active before the embedding volcanic event due to the high amount of well-preserved foliage found around the stem. From the block of KH0196-03, it is reconstructed that fronds have been initially departing from the stem by an angle close to 90° before they were distally bending due to gravitational forces (Fig. 7E).

The attached fronds of *A. schneideri* represent the most complete and largest specimens of this type known so far and simultaneously reveal their definite association to *M. stellata* stems for the first time. Their petioles and rachises show affinity to *Myeloxylon* sp., evidenced by the scattered vascular strands surrounded by sclerenchymatous tissues and the marginal sclerenchymatous plates forming a ring of strengthening elements, visible as parallel striation in adpressions (Fig. 8). The affinity of *Myeloxylon* to medullosans and alethopterid foliage types has long been known (Brongniart, 1849; Grand’Eury, 1877; Rößler & Pönitz, 2006). However, in contrast to the massive shape of most of the known *Myeloxylon*-type petioles and rachises (*e.g*., Renault, 1896), the petioles attached to KH0196 appear distinctly slenderer, and thus tend to have a low potential for anatomical preservation. Therefore, we assume that most of the massive *Myeloxylon* specimens described in the literature were not associated with *M. stellata* but with other medullosans with more prominent petiole bases, such as *M. leuckartii* (Göppert & Stenzel, 1881; Weber & Sterzel, 1896; Luthardt et al., 2021).

The *A. schneideri* fronds of KH0196 represent strictly plagiotropic foliated organs with bilateral symmetry (distichous phyllotaxis) and planarly arranged pinnae (Fig. 11A). They were extending over more than three metres, as evidenced by KH0341, which is estimated to have been ca. 3.56 m long (1.23 m petiole + 2.33 m estimated length of the primary pinnae). For KH0342, a length of 3.54 m (0.93 + 2.61 m) is likely. We suppose that *A. schneideri* fronds had a convexly arcuate shape of primary pinnae, as known from many medullosan frond reconstructions (*e.g*., Laveine, 1997; Laveine & Oudoire, 2009). This shape prevents overlapping of the inner secondary pinnae due to the low bifurcation angle of 45°, thus increasing the photosynthetic efficiency of the leaf surface area. The tripinnate nature of the fronds is deduced from small secondary rachises (≤0.35 m) at the inside and distinctly larger secondary rachises (≤0.75 m) at the outside of the primary rachis. Based on the mean distance between preserved secondary rachises, we assume that up to six of them were attached to the inside and the outside of one primary rachis. Secondary pinnae decrease in size with increasing distance from the stem, which is reconstructed from distally decreasing diameters of basal secondary rachises. Their original attachment angle is supposed to have been 70 to 75°. Foliation of the terminal part of the primary rachises remains speculative but might terminate in a structure similar to secondary pinnae. Specimens TA0512 and TA0517 both impressively show the morphology and architecture of large outer secondary pinnae. Each pinna was densely foliated, leaving practically no gaps between pinnules, pointing to highly efficient use of light. In addition, with dimensions of up to 0.75 × 0.35 m, each pinna exhibited a considerable leaf surface area. The maximum width of a pinna is reached at ca. 3/5 of its length and was rapidly tapering at the tip, where pinnules of tertiary pinnae are fused to form strap-shaped tertiary pinnules. The latter is known as an imparipinnate configuration, which also occurred in other pteridophylls (Laveine, 1997) and is not a specific characteristic of *A. schneideri*. The evolutionary or ecological significance of this configuration remains speculative.

Earlier reports on *A. schneideri* foliage were mainly based on small isolated pinna fragments (*e.g*., Barthel, 1976b; Barthel, 2006). Only a few larger finds from the Chemnitz and Ilfeld basins show some details of the frond architecture. However, compared to the up to 3.5 m long fronds of KH0196, all of these specimens are distinctly smaller, such as the one described from Chemnitz-Hilbersdorf preserved at its level of bifurcation (Sterzel, 1918, Plate 8, Fig. 87). In contrast to KH0342, Sterzel’s specimen further shows a lower order of secondary pinnae attached to the primary rachis, and *Cyclopteris* leaves attached to the petiole. As it was found in the same pyroclastic horizon as KH0196, the question of the reason for these major intraspecific morphological variations arises. We suggest that variations might be best explained by different ontogenetic stages of the same plant species. Thus, the specimen described by Sterzel (1918) might have belonged to a juvenile *M. stellata* plant, whereas KH0196 indeed represents an adult individual. Following this, the cyclopterid foliage of the petiole might describe a character of juvenile *A. schneideri* fronds, as they have not been recognised at the petioles of KH0196. Instead, there is sufficient evidence for smaller secondary pinnae attached to the petioles of adult *A. schneideri* fronds, which is based on foliage found closely related to the petiole of KH0342 and basal rachises attached to some other petioles (KH0341, 343).

Reproductive organs of *A. schneideri* fronds were unknown so far. Some medullosan pollen organs occur together with foliage in the central European Rotliegend Basins, such as *Whittleseya*, *Aulacotheca*, and *Sterzelitheca chemnitzensis* (Barthel & Brauner, 2015), the latter first known from the Chemnitz Fossil Forest (Feng et al., 2014). The structure found attached to the tip of a secondary pinna of TA0509 (Fig. 9E) is interpreted to represent a medullosan pollen organ, which shares many similarities with *Aulacotheca* Halle (Halle, 1933; Stidd, 1981). *Aulacotheca* frequently occurs in strata of Pennsylvanian age and is associated with *Alethopteris* and other medullosan foliage (Stidd, 1981; Laveine, 1997). It also occurs in early Permian strata of central Europe, *e.g*., at the Sperbersbach locality (central Germany, Asselian–Sakmarian), together with *Neurocallipteris planchardii* as the only medullosan foliage type (Barthel & Brauner, 2015). Future preparation work on KH0196 might provide further insights into the reproductive nature of *M. stellata* and *A. schneideri*.

### Growth mechanisms and functionality

Medullosans are supposed to have been growing in various forms, including climbing or scrambling (Krings & Kerp, 1999, 2006; Dunn et al., 2003; Rothwell, 2020), leaning, semi-self-supporting and self-supporting habits (Stewart & Delevoryas, 1956; Wnuk & Pfefferkorn, 1984; Mosbrugger, 1990). Most climbing and scrambling forms are known from Carboniferous coal strata and characterised by small stem diameters and a liana-like arrangement of stem tissues (*e.g*., Dunn et al., 2003). In contrast, medullosans of early Permian strata have distinctly more massive stems and cylindrically arranged secondary tissues, suggesting a self-supporting growth habit for most of these taxa (Mosbrugger, 1990; Luthardt et al., 2021). The *M. stellata* plant of KH0196 is supposed to have been self-supporting, which is deduced from (1) the straight and regular stem stature, (2) the cylindrical arrangement of secondary tissues around the peripheral vascular segment, considerably contributing to stiffness and stability of the stem, and (3) the short internodal distance between adjacent fronds, supposing a slower vertical growth compared to climbing plants such as vines or lianas, which usually show large internodal distances (*e.g*., Krings & Kerp, 2006).

The medullosan crown of KH0196 with its large plagiotropic fronds corresponds to a plant architectural model that is also shared by tree ferns, some extant palms and cycads (Corner’s Model, after Hallé, Oldeman & Tomlinson, 1978). The growth strategy behind this model is to explore the forest space horizontally by large fronds in competition for light in the shaded lower storey. Plants exhibiting this model are usually k-strategists with high individual age and heights not exceeding 15 m and were growing in stable environments of (sub-)tropical forests (Hallé, Oldeman & Tomlinson, 1978). Similar plant architecture is suggested for some medullosans of Pennsylvanian age, such as *M. noei* (Stewart & Delevoryas, 1956) or specimen “G” from Pfefferkorn et al. (1984; Fig. 1). In contrast to these reconstructions, the *M. stellata* var. *typica* crown presented here seems to have had larger dimensions and a somewhat more massive stem.

#### Ontogenetic development

The complex stem anatomy of medullosans and their ontogenetic development are still poorly understood (Luthardt et al., 2021). The medullosan crown of KH0196 is supposed to have been part of an adult specimen. The apex-near transverse sections 1–9 (Fig. 4) provide some new information on primary and secondary meristematic activity of this ontogenetically young stem segment. The number of accessory strands and diameter of the central parenchymatous tissue both remain constant over the whole segment, whereas the overall diameter of the stem slightly increases to its base. The latter coincides with a minor secondary thickening of central accessory strands and the peripheral vascular segment. However, these trends are only apparent in sections 6–9, whereas the measured values remain constant in sections 1–6. Thus, the arrangement and dimensions of anatomical units have developed during apical meristematic activity and remain constant in stems of *M. stellata* var. *typica*. In contrast, lateral meristematic activity and thus secondary thickening of the stem was of minor importance during ontogenesis, resulting in long, tube-shaped stems. The absence of growth rings in the secondary xylem of the vascular system, and accessory strands also indicate subordinated non-seasonal secondary growth. The dominance of apical meristematic activity may result in faster vertical growth of the plant compared to other gymnosperms with secondary thickening and branched stems.

#### Water transport and anatomical organisation of vascular system

Regarding the attachment of ten photosynthetic active, more than three metres long and densely foliated fronds, the leaf surface area of the plant must have been very high, suggesting that the transpiration potential of the plant must have been high, as well, even though the plant was growing in the more shaded lower storey. This assumption is supported by hypostomatic cuticles of *A. schneideri* from its type locality (Sterzel, 1881), which reveal a high stomata density (M. Barthel, 2016, personal communication). High transpiration rates necessitate a high water flow capacity of the stem tissues. In KH0196, water transport is supposed to have been realised by both primary and secondary xylem of the peripheral vascular system. Metaxylem (≤250 µm wide) and secondary tracheids (⌀ 73 µm wide) of KH0196 are large compared to other gymnosperms and were potentially able to transport increased quantities of water and derivates (*e.g*., Wilson et al., 2008). The system of central accessory strands most probably provided additional conducting capacities, even though its connection to the peripheral vascular system and fronds remains unclear (Luthardt et al., 2021). As sections 7–9 (Fig. 4) show, they might be connected to emerging plant organs of unknown affinity. Fronds are connected by several leaf traces (>9) arising from the primary xylem of the peripheral vascular segment. The leaf traces divide by an unclear organisation mode into numerous vascular strands in the basal petiole. From sections 7–9, it might be referred that leaf traces are merged to form a single wing-shaped vascular element from which petiole vascular strands are dividing. The oval gap in the peripheral vascular segment associated with attached petioles enables a connection of the central parenchymatous tissue and the cortex parenchyma but shows no outgoing conducting elements. Its significance remains speculative.

### Palaeoecology and stratigraphic implications

*Alethopteris schneideri* represents a morphologically characteristic form that can be clearly distinguished from other pteridophylls of the Rotliegend flora, even though it exhibits certain morphological variations (Barthel, 1976b; Barthel, 2006). It represents a typical element of the Lower Rotliegend flora (Table 1) and is widely known from most of the intramontane basins of central and western Europe (Barthel, 1976b; Wagner & de Sousa, 1983; Wagner & Álvarez-Vázquez, 2010), but also of North America (New Mexico, DiMichele et al., 2013). *Alethopteris schneideri* and *M. stellata* are absent in peat-forming forest communities (Barthel, 2006) and those of poorly-drained clastic soils, as has been supposed for *M. stellata* in the Chemnitz Fossil Forest (Luthardt et al., 2021). Nevertheless, its preferred affinity to habitats on moist clastic soils with near-surface groundwater levels is suggested from all fossil localities, mostly located in basin-central regions and/or in proximity to lake environments. Thus, *M. stellata/A. schneideri* must be regarded as a hygrophilous–mesophilous plant taxon of lower Permian seasonally-dry forests.

**Table 1:** Stratigraphic and palaeo-environmental occurrence of *M. stellata* stems and *A. schneideri* foliage.

Locality	Plant organ	Stratigraphy	Sed. facies/ Palaeo-environment	Reference
Chemnitz Basin (Germany)	*M. stellata* var. *typica* *A. schneideri*	Härtensdorf–Leukersdorf Fm., Asselian–Artinskian	alluvial plain, clastic soil, near-surface groundwater level, seasonal sub-humid palaeoclimate	Cotta, 1832 Weber & Sterzel, 1896 Luthardt, Rößler & Schneider, 2016, Luthardt et al., 2021
Northwest Saxony Volcanite Complex (Germany)	*M. stellata* var. *typica* *A. schneideri*	Asselian–Sakmarian	unknown	Cotta, 1832 Sterzel, 1881 Barthel, 1976a
Ilfeld Basin (Germany)	*M. stellata* var. *typica* *A. schneideri*	Upper Pennsylvanian–lower Permian	unknown	Sterzel, 1901a, 1901b Barthel, 1976a
Döhlen Basin (Germany)	*A. schneideri*	Niederhäslich Fm., Sakmarian	paralic, peaty substrate?	Barthel, 1976a
Thuringian Forest Basin (Germany)	*A. schneideri*	Manebach Fm., Goldlauter Fm., Oberhof Fm. Asselian–Sakmarian	clastic soils, lacustrine proximity (absent in peat floras)	Barthel, 2006; Barthel & Brauner, 2015
Saar-Nahe Basin (Germany)	*A. schneideri*	Meisenheim Fm., Asselian–Sakmarian	unknown	–
Nová Paka, Krkonoše Piedmont Basin (Czechia)	*M. stellata* var. *typica*	Semily Fm., Gzhelian–Asselian	alluvial plain, wet clastic soil, lacustrine proximity, sub-humid palaeoclimate	Opluštil et al., 2013
Boskovice Basin (Czechia)	*A. schneideri*	Padochov Fm., Asselian	bituminous carbonate (Zbýšov Horizon)	Lipps, 1927 Opluštil et al., 2013
Autun Basin (France)	*M. stellata* var. *typica* *Alethopteris* sp.	Millery-Fm., Asselian–Sakmarian	alluvial plain, fluvial proximity?, (sub-) humid	Renault, 1875
Southern Alps (Italy)	*Alethopteris* foliage *with aff. to A. schneideri*	Gröden Sandstone, Lopingian	unknown	Kustatscher et al., 2014
Buçaco (Portugal)	*A. schneideri*	unknown	unkown	Wagner & de Sousa, 1983
Kinney Quarry (New Mexico, U.S.A.)	*A. schneideri*	Gzhelian (Upper Pennsylvanian)	coastal lagoonal setting	DiMichele et al., 2013

The growth-architecture model of *M. stellata* is well-adapted to the more shaded lower storey of the forest habitat. The high leaf surface area, impressively shown by the ten active, up to 3 m long fronds of KH0196, in combination with the supposed high water flow capacity of the stem tissues, suggests a considerable transpiration potential of the plant. Thus, the availability of soil water throughout the year might have played an essential role for *M. stellata*. Nevertheless, a certain drought resistance could be indicated by the slightly revolute pinnule margins and the hypostomatic epidermis structure of *A. schneideri* (Reichel & Barthel, 1964).

Stems and foliage of the *M. stellata*/*A. schneideri* plant both occur in a restricted stratigraphic range. Thus, the taxon can be seen as a stratigraphic relevant element in the intramontane European Rotliegend Basins (Asselian–Artinskian, lower Permian), even though *A. schneideri* first occurred in the late Gzhelian (uppermost Carboniferous; DiMichele et al., 2013). Its stratigraphic last occurrences in the Millery Fm. (Autun Basin), Leukersdorf Fm. (Chemnitz Basin) and Oberhof Fm. (Thuringian Forest Basin) around the Sakmarian–Artinksian boundary could be either related to a large taphonomic bias in the middle–late Permian sedimentary record or to the disappearance of the wet basin-central ecosystems at this time. The occurrence of alethopterid foliage with potential affinity to *A. schneideri* from the Lopingian strata of the Southern Alps (Kustatscher et al., 2014) might indicate that this medullosan taxon survived up to the latest Permian in ecological refugia.

## Conclusions

1. We present the first comprehensive crown reconstruction of the early Permian seed fern *Medullosa stellata* var. *typica*, based on organic connections of the anatomically preserved upper stem and foliated fronds of the *Alethopteris schneideri* type, predominantly preserved as adpressions.
2. The stem has wide central parenchymatous tissue with up to 87 accessory strands and a peripheral, cylindrical vascular segment with centripetally and centrifugally grown secondary tissues. Where petioles were attached, the peripheral vascular segments typically show openings, and many leaf traces emerge from the primary tissue of the peripheral vascular segment. Stem secondary tissues are produced during an early ontogenetic stage, whereas lateral meristematic activity is low in later ontogenetic stages. Compared with other gymnosperms, the stem vascular system is inferred to have had a high water transport capacity.
3. *Alethopteris schneideri* fronds have a petiole and rachises of the *Myeloxylon* type exhibiting numerous vascular strands and secretory canals. Fronds were plagiotropic and of determinate growth. They were up to 3.5 m long, bifurcating and exhibited alternating secondary pinnae of up to 0.8 m length to both sides of the primary rachises, each bearing densely arranged tertiary pinnae of up to 0.17 m lengths.
4. The here investigated specimen had up to ten active, densely arranged fronds, showing a 3/8 phyllotaxis. Older, died off fronds are absent and were most probably abscised shortly after becoming inactive.
5. *Medullosa stellata* was a self-supporting plant of straight and slender stature, well adapted to grow in the light deficient lower storey of seasonally-dry forests dominated by large cordaitalean trees.
6. Based on the fossil record of *A. schneideri*, the *M. stellata* plant is a stratigraphically characteristic meso- to hygrophilous element of the central European Rotliegend flora (Asselian–Artinskian, lower Permian), preferably growing on moist to wet clastic substrates and sub-humid, seasonal palaeoclimatic conditions.

## Supplemental Information

10.7717/peerj.13051/supp-1 Supplemental Information 1 List of specimens used to reconstruct the Medullosa crown.

10.7717/peerj.13051/supp-2 Supplemental Information 2 Primary data on stem anatomical quantitative characteristics.

10.7717/peerj.13051/supp-3 Supplemental Information 3 Primary data on frond architecture measured on fossils.

## Additional Information and Declarations

### Data Availability

The following information was supplied regarding data availability: The quantitative data is available in the Supplemental Files.
