# Supplementary material for: Upside-down in volcanic ash: crown reconstruction of the early Permian seed fern Medullosa stellata with attached foliated fronds
Source: PeerJ. 2022 Mar 22;10:e13051. doi: 10.7717/peerj.13051 (PMC8953532; doi:10.7717/peerj.13051)
Supplement: Supplemental Information 1 [file peerj-10-13051-s001.docx]

**Specimen list of Medullosa crown and additional information**

Specimen-No.: KH0196

Taxon: Medullosa stellata var. typica Cotta 1832

Name: “Upsidedown-Medullosa”

Data point protocol number: DP0027

Protocol number: FP0134

Photography number: FO0210

KH0196-01,-02,-03 (stem)

KH0338 (frond)

KH0339 (frond)

KH0340 (frond)

KH0341 (frond)

KH0342 (frond)

KH0343 (frond)

KH0344 (frond)

KH0345 (frond)

KH0346 (frond)

KH0347 (frond)

KH0348 (frond)

TA0478 (FP0134, FO0210, DP0055)

TA0508 (Area-TA, S5P-029, FO0422, DP0028)

TA0518 (Area-TA, S5P-029, FO0422, DP0029)

TA0524 (Area-TA, S5P-039, FO0439, DP0030)

TA0517 (Area-TA, S5P-034, FO0422, DP0049)

TA0519 (Area-TA, S5P-034, FO0422, DP0050)

TA0520 (Area-TA, S5P-034, FO0422, DP0051)

TA0521 (Area-TA, S5P-034, FO0422, DP0052)

TA0522 (Area-TA, S5P-034, FO0422, DP0053)

TA0512 (Area-TA, S5P-033, FO0352, DP0054)
